# Multimodal neuroimaging of male and female brain structure in health and disease across the life span

**DOI:** 10.1002/jnr.23919

**Published:** 2016-11-07

**Authors:** Neda Jahanshad, Paul M. Thompson

**Affiliations:** ^1^ Imaging Genetics Center, Mark and Mary Stevens Neuroimaging & Informatics Institute, Keck School of MedicineUniversity of Southern CaliforniaMarina del ReyCalifornia

**Keywords:** brain development, sexual dimorphism, neuroimaging, magnetic resonance imaging, white matter

## Abstract

Sex differences in brain development and aging are important to identify, as they may help to understand risk factors and outcomes in brain disorders that are more prevalent in one sex compared with the other. Brain imaging techniques have advanced rapidly in recent years, yielding detailed structural and functional maps of the living brain. Even so, studies are often limited in sample size, and inconsistent findings emerge, one example being varying findings regarding sex differences in the size of the corpus callosum. More recently, large‐scale neuroimaging consortia such as the Enhancing Neuro Imaging Genetics through Meta Analysis Consortium have formed, pooling together expertise, data, and resources from hundreds of institutions around the world to ensure adequate power and reproducibility. These initiatives are helping us to better understand how brain structure is affected by development, disease, and potential modulators of these effects, including sex. This review highlights some established and disputed sex differences in brain structure across the life span, as well as pitfalls related to interpreting sex differences in health and disease. We also describe sex‐related findings from the ENIGMA consortium, and ongoing efforts to better understand sex differences in brain circuitry. © 2016 The Authors. Journal of Neuroscience Research Published by Wiley Periodicals, Inc.

## 1 INTRODUCTION

Worldwide, there are sex differences in the prevalence of many major psychiatric and neurological disorders across the life span. Autism spectrum disorder (Fombonne, 1999; Werling and Geschwind, 2013) and schizophrenia (Ochoa et al., 2012), for example, are consistently more prevalent in boys or young men. Other disorders are more prevalent in women than men, including major depressive disorder (Weissman et al., 1984) and anorexia nervosa (Striegel‐Moore et al., 2009); many other disorders, including bipolar disorder, present little or no difference in primary rates, though rates of comorbid conditions and consequences may vary (Weissman et al., 1984; Diflorio and Jones, 2010). These sex differences in disease prevalence continue throughout adulthood, and disorders such as major depression are common. There are also sex differences in the risk factors, average age of onset, and prevalence of late‐life dementias, as well as cerebrovascular disease (Riedel et al., 2016).

The neurobiology and risk factors for many of these diseases are still not well understood. Sex differences in brain development and aging are vital to identify, as they may point to mechanisms or biological processes that lead to sex differences in disease risk and potential treatment (Cahill, 2006). Sex differences in the brain and behavior can arise due to many factors; there are fundamental differences in the genome between men and women, including differences in the X and Y sex chromosomes, which harbor a number of disease‐related genetic loci. There are also sex‐specific genetic programs, such as those that regulate the hypothalamic–pituitary axis and endocrine system as a whole. The endocrine system also interacts dynamically with the brain and behavior throughout life. Epigenetic changes also occur, and these in turn depend on a person's genetics and environment, and they may also lead to fundamental differences in brain organization and function (McCarthy et al., 2009).

Despite known neurobiological and molecular differences, many claims of sex differences in brain structure are controversial or disputed, and are therefore important to evaluate objectively.

Human brain imaging has been used for decades to study sex differences in brain structure and function, often yielding new hypotheses. At the time of writing, a Google Scholar search of ‘neuroimaging and “sex differences”’ reports 16,900 scholarly publications, in the last five years alone (January 2011–May 2016). Over 300 of these articles are indexed in PubMed, and more than two dozen are review articles. With on average five new and independent review articles focused on neuroimaging correlates of sex differences per year, over the past five years, there is clearly widespread interest in the field; yet there is still no consistent or agreed set of findings. In fact, a recent publication that has received much debate performed a large, multisite, structural brain imaging study of sex differences (Joel et al., 2015). Consistent “male” or “female” structural patterns across regions were hard to identify. When evaluating the regions independently, they found that effect sizes for sex are only moderate for the most differentiable brain regions and imaging measures; even so, there may be more complex neuroanatomical patterns that differ by sex that are not easy to distinguish from structural brain images or by evaluating single measures one at a time (Rosenblatt, 2016). It should be noted that Joel and colleagues’ work studied young adults aged 18 to 28. This period includes the later stages of brain maturation, and results may therefore be affected by differences between male and female developmental trajectories, which were not assessed.

As is common in surveys of this kind, we begin with a caveat that group differences in the brain and behavior may not indicate trait values in any individual woman or man; secondly, we must be aware of some confounds in both imaging and epidemiology that affect how sex differences are interpreted, and that can help in understanding some controversies in the field and why they arose.

In this review, we highlight some major findings in the imaging literature that point to sex differences, on average, in brain structure and in the brain's path through life. The complexity of the brain, and its many individual and connected components—along with the numerous neuroimaging features that can be extracted—have led to thousands of reports of sex‐related differences. Here, we briefly discuss the evidence for sex differences in gross anatomy, identifiable with neuroimaging, highlighting key publications. For most of this review, we focus on one of the most widely studied structures in the human brain, the corpus callosum—the major white matter fiber bundle connecting the left and right brain hemispheres. For an extensive review of neuroimaging studies of the corpus callosum, please see Thompson et al., 2003 2003. The corpus callosum is strongly implicated in a variety of sex‐linked genetic disorders including supernumerary sex chromosome aneuploidies (Wade et al., 2014) and Fragile X syndrome (Villalon‐Reina et al., 2013), as well as many other complex neuropsychiatric disorders ranging from major depressive disorder (Ballmaier et al., 2008), bipolar disorder (Fears et al., 2014), and schizophrenia (Narr et al., 2000; Kochunov et al., 2014), to various types of dementia (Daianu et al., 2015), and many others. Not only is it implicated in these numerous diseases with sex differences in their prevalence, it is also highly genetically influenced (Bearden et al., 2011; Jahanshad et al., 2013; Kochunov et al., 2016). More practically, unlike some other structures with widely reported structural and functional sex differences, like the amygdala (Hamann, 2005), the corpus callosum is simple to extract and measure from many modalities of neuroimaging; given the controversy in findings, a structure that is easier and more reliable to measure (Morey et al., 2010) might be a good target to analyze, and methodological errors in its measurement are perhaps less likely to influence the reported sex differences.

We organize this review by first summarizing work on sex differences in the development and aging of the normal brain, first in terms of overall volume and callosal size. We also discuss studies using more advanced metrics of white matter microstructure and brain connectivity derived from imaging modalities beyond standard structural magnetic resonance imaging (MRI), such as diffusion‐weighted MRI (dMRI), a now prevalent noninvasive MRI technique that allows for the modeling of the magnitude and direction of water diffusion in the brain, revealing microstructural properties of the myelinated white matter. We discuss known and hypothesized brain correlates of the sex differences in psychiatric and neurological disease risk and outcomes. We conclude by pointing to value of global neuroimaging and genetics consortia, such as the Enhancing Neuro Imaging Genetics through Meta Analysis (ENIGMA) consortium (Thompson et al., 2014), for evaluating and testing claims of sex differences. As will emerge from our review, a key future objective is understanding the reproducibility and generalizability of sex differences across populations. This is crucial for discovering what factors drive healthy and disease‐associated sex differences.

## 2 SEX DIFFERENCES IN BRAIN STRUCTURE OVER THE LIFE SPAN

Before considering brain development and aging, and their dynamic effects throughout life, we examine the debate that has surrounded sex differences in brain structure in young healthy adults. In this field, a number of caveats are useful to understand claims of sex differences or better understand what is driving them.

### 2.1 Height‐ and Brain‐Size‐Driven Sex Differences

In many anthropometric studies—and the brain is no different—it is important to take into account differences in body size and how they may interact with the features being measured. In most Western societies, adult men are, on average, taller and heavier than young adult women. As people with large bodies tend to have larger heads, and larger brains, it is not surprising that the average brain size of adult men is larger overall. Early neuroimaging studies reported that the mean volume of the forebrain is about 9% higher in young adult men than in age‐matched women (men: 1.08 ± 0.11 liters (*n* = 71, age: 25.3 ± 4.6), women: 0.99 ± 0.10 liters (*n* = 49, age: 26.3 ± 4.9) (Jäncke et al., 1997). The average intracranial volume (ICV) in adult males is around 9% to 12% larger than that of adult females (Lenroot and Giedd, 2010; Giedd et al., 2015). One remarkable claim is that, on average, the volume of the male brain before the age of 6 is larger than that of the average adult female (Giedd et al., 2015). A similar 10% sex difference in mean postmortem brain weight was reported long before imaging studies were common (Voigt and Pakkenberg, 1983).

While it is imperative to take into account these global differences, the sizes of individual parts of the brain do not just scale linearly with the height of the person, or with the overall size of their brain.

We therefore need to consider what aspects of the reported sex differences in brain measures are simply due to the overall size of the person, vs. more specific biological processes underlying fundamental differences in brain structure and function.

Comparative volumetric studies using quantitative MRI took off in the 1990s; these initially segmented the MRI scan into the two major tissue types: gray matter, consisting of neuronal cell bodies, dendrites, synapses, and axon terminals; and white matter, consisting of myelinated axons interconnecting different gray matter regions. Greater white matter volume in men was initially thought to be driving the differences in overall volume (Filipek et al., 1994; Passe et al., 1997), with reports of an increased proportion of gray matter in women (Gur et al., 1999). By the late 1990s, detailed computational maps of the brain were allowing substructures within the brain to be compared on a quantitative level between sexes (Gur et al., 1999) and tested for correlations with cognitive measures.

These initial papers led to reports in the popular press that speculated how sex differences in size and shape of localized brain regions might account for some differences in behavior, cognition, disease risk—and even disease outcomes—between women and men. However, since these initial reports of differences in overall gray matter and white matter volumetric measurements, inconsistencies have plagued the thousands of papers on sexual dimorphisms in brain structure on MRI (for a recent meta‐analysis, please see Ruigrok et al., 2014). Conclusions were sometimes drawn from inconclusive evidence, or from small samples; in other cases, studies failed to account for overall height or brain size, or did so in different ways. Given the initial findings of white matter differences, the cross‐sectional size of the corpus callosum (CC)—an easy feature to measure—became a natural region of interest and the focus of many conflicting papers. An extensive review of these findings and the inconsistencies reported was conducted by Thompson et al. (2003 2003).

### 2.2 Brain Size Adjustments

In an attempt to factor out the effects of gross variations in brain size, early studies of sex differences in the corpus callosum used the *ratio* of total callosal area to a measure of whole brain size. Even so, ratio measures do not fully adjust for brain size. Many standard statistical models, such as linear regression or analysis of covariance, typically assume that callosal measures vary linearly with extraneous parameters such as brain size. If these models are used, differences persist that are still due to differences in brain size—in other words, a group of tall women, compared with short men, would show the opposite pattern of differences.

Jäncke et al. (1997) suggested that a log‐linear relation, or “power law,” exists in adults between the total callosal area (Area_CC) and forebrain volume (FBV). This can be modeled roughly as Area_CC = constant × (FBV)^*p*^. Their estimates of the power *p* (0.66 in females, 0.52 in males) were based on significant regressions in an MRI cohort of 120 subjects. Since callosal area increases less than linearly with forebrain volume (i.e., *p* < 1), and as women have smaller brains on average, the ratio of total callosal size to brain volume will automatically be larger in women in the absence of other factors. This indicates a general brain size effect, independent of sex, so that the altered proportion is not a specialized feature of the structure being analyzed (Clarke et al., 1989). As a brain size correction, the *p*th power of FBV, estimated empirically from the sample, is likely to provide a useful covariate for multivariate statistical tests, if removal of brain size effects is required. However, an even more general model might be required if the value of the exponent *p* were also found to depend on sex. As a result, sex differences in any proportional measures (such as “splenial area as a proportion of total callosal area”) must be interpreted with caution, as any association between sex or age and the ratio's denominator can create a substantial effect (Sowell et al., 2007). Luders et al. (2014) compared callosal dimensions between men and women deliberately matched for overall brain size, to clarify the true contribution of biological sex. They concluded that hardly any callosal differences remained between brain size–matched men and women, but that larger samples might inform us further on the subtle effects that still remained. This does not mean that there are not more complex patterns of difference in the images, just that misleading interpretations of the current data may be made if these adjustments are not performed or understood.

Recent works have suggested that methodological considerations may be to blame for widespread inconsistencies even beyond the corpus callosum, including subcortical structures and regional cortical volumes. When studies properly take into account the adjustment for ICV, some of the localized differences in volume of subcortical structures are no longer observed, although different correction methods give different results (Nordenskjold et al., 2015; Pintzka et al., 2015).

Offering more detailed brain metrics than gross morphometry, brain mapping techniques have expanded beyond the ability to compare volumes, to finer details of microstructure, such as the structural connections that can be mapped with dMRI.

*Diffusion MRI*. dMRI, including diffusion tensor imaging (DTI), can be used to investigate fine‐scale details of the white matter structure, beyond size or volume. Scalar measures from dMRI, including diffusivity and anisotropy, offer quantitative metrics of fiber organization, axonal myelination, and fiber density.

Of the scalar measures derived from dMRI, perhaps the most common are fractional anisotropy (FA) and mean diffusivity, which, compared with regional volumes, are relatively insensitive to intracranial volumetric differences (Sullivan and Pfefferbaum, 2003), although positive relationships have been reported between overall FA and ICV (Takao et al., 2011). More regionally, differences in white matter tract size may lead to systematic differences; if the size or volume of a tract is greater in males, then the females may be more prone to partial voluming effects that affect measurements in the relatively large dMRI voxels, artificially reducing the anisotropy measures (Jones and Cercignani, 2010). To ensure the sex differences in white matter microstructure were independent from callosal volumes, Westerhausen et al. (2011) evaluated correlations between callosal volume and FA, and also FA differences between the sexes in the corpus callosum. They reported higher FA in men, consistent with most other reports across many regions of the CC. However, only in anterior parts of the genu of the corpus callosum, where sex differences were identified, did they note that FA did not correlate with callosal size. This suggests that sex differences, at least in localized regions of the CC, may well be due to microstructural differences—not driven by systematic volume‐induced errors.

### 2.3 Life Span Trajectories of Brain Development and Aging

More in‐depth studies of sex differences in developmental trajectories became possible when pediatric imaging studies scanned larger cohorts. One of the first such studies, led by Dr. Judith Rapoport at the National Institute of Mental Health, scanned hundreds of children, between infancy and adulthood, and led to the first comprehensive reference data on brain development as seen through imaging. In one of the most highly cited studies in pediatric imaging (Gogtay et al., 2004), a picture emerged in which the brain's development follows a stereotypical sequence—the earliest to mature are the more primitive brain regions involved in sensation, vision, and touch, and higher‐order brain regions involved in language and executive function tend to mature later, with ongoing changes in all brain regions proceeding well into adolescence and beyond.

In one of the largest developmental studies of sexual dimorphism, Lenroot et al. (2007) scanned 387 individuals aged 3 to 27, multiple times, acquiring over 800 MRI scans in total. The *sequence* of development was largely identical when average trajectories were created for boys and girls. This was a remarkable finding at the time, as some had hypothesized that there was a greater hemispheric asymmetry in the male brain, and that inevitably, that must lead to an observable sex difference as such asymmetries tend to increase with age (Sowell et al., 2002). As with puberty, some brain changes occurred around 1.5 to 2 years earlier in girls than boys, with boys “catching” up soon after puberty. In other words, the sequence of development was strikingly similar, but somewhat earlier in girls. White matter volume increases were seen throughout the observed age range; and, without adjusting for head size, trajectories diverged between the sexes, as males increased faster during adolescence. They did report sex differences in the age‐matched size and trajectory of most brain regions they evaluated, based on the height and shape of the developmental curves. Even so, no significant differences were seen in the area of the midsagittal corpus callosum until they adjusted for total brain volume; and then, only the height of the curve, not the shape, was different, implicating larger CC areas in females.

Developmental differences between the sexes have been studied with DTI during adolescence both cross‐sectionally (Asato et al., 2010) and longitudinally (Simmonds et al., 2014). Analyzing data from 128 individuals scanned up to five times annually, Simmonds et al. found that in general, female—as measured with DTI—matures primarily during adolescence, while the white matter microstructure of male counterparts tends to mature from childhood through early adulthood. They found significant age‐by‐sex developmental differences for white matter microstructure overall. After breaking down the white matter into regional measures, no differences were detected in the developmental trajectories of the corpus callosum or its components, but there were sex differences in limbic and cerebellar regions; callosal developmental trends in size (Lenroot et al., 2007) and diffusion‐based microstructure (Simmonds et al., 2014) showed similar trends between sexes in healthy development. Age‐by‐sex interactions continue to be reported in late adulthood as well. Some studies find faster rates of brain decline in men (Kochunov et al., 2012), but not all studies detect differences (Bartzokis et al., 2012).

Brain connections can also be mapped with dMRI using tractography, where the directionally constrained diffusion signal is traced from voxel to voxel throughout the brain, mapping out representations of the white matter connections. The brain's structural “connectome”—or connectivity pattern—may then be mapped by combining tractography from dMRI with the more commonplace cortical segmentations based on high‐resolution T1‐weighted MRI, and determining properties of the connections between pairs of cortical regions. The connection “strength” may be defined based on numerous factors including a simple count of “streamlines” (neural pathways detected by the tractography algorithm) or FA along the streamlines. Sex differences in the connectome have been reported. In a large cross‐sectional study of brain connectivity across adolescence and adulthood, Ingalhalikar et al. (2014) found the “strength” of connections within a hemisphere to be greater in men, and the strength of interhemispheric connections (such as those running through the corpus callosum) was greater in women. Despite added methodological variability when combining two modes of imaging, prior independent studies reported similar findings with respect to average intra‐ and interhemispheric connection differences between the sexes (Jahanshad et al., 2011).

Connectomes may also be viewed as topological graphs or networks. Global network measures can describe topological properties such as network efficiency or clustering; other measures evaluate the integration or segregation of the nodes. In general, these measures describe more the overall patterns of connections at one node or across all nodes in the network, and not the inter‐ or intrahemispheric specializations, but these topological measures are sexually dimorphic (Gong et al., 2009) and are hypothesized to account for some of the normal variance in behavioral or cognitive measures. In Duarte‐Carvajalino et al. (2012), these topological measures were found to be good predictors of the sex of the individual. In recent work, researchers used the same dataset as Ingalhalikar et al. (2014) to identify possible functional and behavior assessments corresponding to the sex differences in connectivity networks (Tunc et al., 2016); they report higher connectivity in males in motor, sensory, and executive function subnetworks and higher connectivity in females in reward and memory subnetworks.

### 2.4 Differential Susceptibility to Disease

The prevalence of many neurological and psychiatric disorders is remarkably different across the sexes. The previous sections of this review focused more on sex differences at large, in the general population, and across the developmental trajectory. As reported, effects are subtle to moderate in the general population, and large sample sizes are needed to obtain statistical evidence of differences, especially when findings are inconsistent or effects are weak. However, most diseases are not exclusive to a particular sex, but simply more prevalent to different degrees. As the brain operates through networks of connections, a multivariate approach to combine measures across neuroanatomical regions may yield better predictors of sex differences in behavior, cognition, and disease.

In addition to the more commonly young‐adult‐onset psychiatric disorders mentioned previously such as schizophrenia, major depressive disorder, and anorexia nervosa, sex differences in prevalence are also widely reported in behavior and cognitive disorders that occur during early childhood as well as in neurodegenerative disorders that tend to occur much later in adulthood.

Autism has at least a two‐ to threefold greater prevalence in males than in females (Fombonne, 1999; Kim et al., 2011; Baxter et al., 2015; Christensen et al., 2016). A reported “female protective effect” has been hypothesized, as X chromosome–linked genetic mutations may be less harmful in females with two copies of the X chromosome, and they may need a greater load of these variants for the disorder to manifest (Baron‐Cohen et al., 2011; Robinson et al., 2013). Increasingly, the behavioral characteristics and functionalities of the “male brain” have been cited as risk factors and characteristics of autism spectrum disorders (Baron‐Cohen et al., 2005). Imaging studies separately comparing boys and girls with autism vs. those without autism are now being conducted, and suggest sex‐specific structural network disruptions underpinning the neurobiology of the disorder (Lai et al., 2013, Retico et al., 2016).

Brain disorders with ages of onset in mid‐ to late adulthood often are neurodegenerative, and include the dementias. While several extensive studies and reviews have been conducted on sex differences in prevalence and cognitive abilities in dementias, including but not limited to Ruitenberg et al. (2001), Li and Singh (2014), and Laws et al. (2016), there are several caveats that need to be considered when exploring group differences later in life. These can include risk of other comorbidities or risk factors, from psychiatric conditions including depression, which has been associated with Alzheimer disease (AD), to cardiovascular health, which also has been associated with AD, stroke, and other later‐life complications (Li and Singh, 2014). Sociological and population biases in study participation and recruitment have also been suggested as a caveat in the interpretation of findings (Hua et al., 2010). Despite the caveats, understanding sex differences in dementia, or dementia risk factors that may be modulated by sex, is an important step to early diagnosis and intervention; it is also important to evaluate these risk factors in the context of healthy aging in both men and women. In a large cohort of over 1200 cognitively normal adults aged 30 to 95, Jack and colleagues (2015) studied AD risk factors, including sex and APOE4 genetic risk, and imaging hallmarks of AD including hippocampal volume and positron emission tomography–based measures of amyloid deposition. They found no difference in amyloid deposition but did observe sex differences in memory scores across the age range, as well as differences in adjusted hippocampal volume. This suggests the importance of making comparisons with sex‐specific norms when evaluating signs of cognitive impairment. Within each sex, they found no evidence that APOE4 carriers showed differences from noncarriers in terms of memory performance or hippocampal volume (Jack et al., 2015). Other than *APOE*, the effects of most known genetic variants have been shown to explain less than 1% of the overall variance in brain traits evaluated so far (Hibar et al., 2015), and large consortium studies of over 10,000 individuals may be needed to detect specific genetic effects common across or perhaps particular to each sex.

### 2.5 ENIGMA, Meta‐Analysis, and Replication

The ENIGMA consortium incorporates over 15 disease‐specific working groups, pooling together case/control effect sizes for differences in regional neuroimaging measures. Through harmonized image processing and worldwide collaboration, the working groups have reproducibly identified disease effects on different parts of the brain; often, prior findings—as in studies of sex differences—had been inconsistent or inconclusive because of small effects on single regions of the brain. Follow‐up investigations on discovered effects are conducted through secondary analyses or meta‐regression approaches to determine whether effect sizes are influenced by moderating factors such as age, medication, or the sex of the individuals in the pooled cohorts. Such meta‐analytic approaches also allow us to assess any differences in effect sizes not only due to the population under study, but due to image acquisition differences across scanners and protocols. Pooling effect sizes across such acquisition protocols and study designs, in a harmonized fashion, helps overcome challenges when comparing findings from discordant published studies. To date, four such studies have been published evaluating subcortical volumes in healthy controls compared with patients, in schizophrenia, bipolar disorder, obsessive compulsive disorder, and major depressive disorder. These ENIGMA studies have not found sex‐by‐diagnosis effects on brain volumes in schizophrenia (van Erp et al., 2016) or within volumes or cortical measures in major depressive disorder (Schmaal et al., 2016a, 2016b). However, in *bipolar disorder* there appear to be significant sex‐by‐disease interactions in the volume of the thalamus, with higher volumes in female patients (Hibar et al., 2016); further work is needed to identify any clinical effects related to this thalamic volume increase.

The ENIGMA consortium is broadening the disorders it studies, bringing together researchers and scientists studying many more conditions with sex differences. A study of substance use across the life span is under way, in unprecedented sample sizes. There may be brain morphometry differences between male and female methamphetamine users (Kogachi et al., 2016), and vulnerability to substance use may be influenced by sex‐dependent developmental trajectories (Hammerslag and Gulley, 2016). A newly formed working group on anorexia nervosa, a disorder more prevalent in women but that also affects men, has recently pooled together numerous cohorts of approximately 20 patients and 20 controls to reach pooled samples of several hundred individuals from around the world. Most of the patients are female within each cohort, but the pooled sample size may for the first time allow for the study of anorexia and its effects and correlates in the male brain.

The consortium is not only evaluating differences in subcortical volumes, but also sex‐by‐disease interactions with respect to cortical thickness and surface area, and measures derived from diffusion imaging. Work is also under way to evaluate the effects of diseases separately on the brain structure of men and women to examine the evidence for differential patterns of brain structure and connectivity networks.

### 2.6 Sex‐Specific Heritability and Sex Chromosomes: Do Genetics Play a Differential Role?

In a large population study of healthy adults, using high‐resolution diffusion MRI from the Human Connectome Project, Kochunov et al. (2015) determined the proportion of variance in FA that is explainable by additive genetic factors, after covarying for age, sex, and their linear and nonlinear interactions. Sex alone was the only significant predictor of FA in young adults, for most regions of the FA skeleton (it was not a significant predictor in two smaller regions). FA was higher in women than in men, by approximately 2%. Heritability is defined as the proportion of variance in a trait attributable to additive genetic effects in a given population. While both sexes exhibited significant genetic influences on average FA as a whole, a greater proportion of the variance in men—91.5% compared with 85.7%—was attributable to additive genetic factors. Men also showed a greater proportion of variance attributable to linear and quadratic effects of age than women (1.5% compared with 0.15%), suggesting perhaps greater unexplained variance overall in the women in this population.

The role of sex chromosomes themselves, as well as sex hormones, are also topics of great interest. A recent study of white matter microstructure evaluated women with complete androgen insensitivity syndrome (CAIS), who lack androgen action in the presence of a 46,XY karyotype (van Hemmen et al., 2016). Affected individuals are phenotypically female and have a female gender identity, despite having a Y chromosome; however, affected individuals often have primary amenorrhea and other reproductive complications (Oakes et al., 2008). The syndrome therefore offers an opportunity to study the role that sex hormones play in comparison with genetic sex on the brain. As in other studies, van Hemmen and colleagues found that their control male group had higher FA than their control female group, and their CAIS female group had FA values most similar to 46,XX female controls, and significantly lower than 46,XY males, suggesting a significant hormone effect, or interaction, over that of sex chromosomes. Interestingly, axial diffusivity measures in those affected were also significantly different from both comparison men and women. Further insight into these findings is needed, as are replication studies.

Recent large‐scale genome‐wide association studies by global consortia such as ENIGMA and CHARGE have pooled genetic association data from up to 30,000 individuals with brain scans and DNA. For the first time, these genomic studies have identified common genetic variants that individually explain approximately 0.5% to 1% of the variance in specific brain structure volumes, in a pooled sample of both men and women (Hibar et al., 2015). Both ICV and sex effects were controlled for in this analysis, aiming to identify genetic variants that affect brain structure irrespective of sex; however, the success of the effort has launched numerous substudies. Ongoing efforts now evaluate genome‐wide associations in men and women separately, to determine any statistical differences in effect sizes in genetic influences on brain structure.

## 3 CONCLUSIONS AND FUTURE WORK

Many psychiatric and neurological disorders have different prevalence, age of onset, and clinical presentation in males compared with females; even so, the biological mechanisms and the neurological risk factors, including those that differentially impact the sexes, are not well understood. Sex differences in brain structure detected with structural neuroimaging may be less prominent than initially believed, particularly after correcting for differences related to height and overall head or brain size. However, that is not to say that important differences do not exist. Small to moderate effects are repeatedly reported across neuroimaging measures. A focus on specific, easily identifiable brain regions, such as the corpus callosum, may help ensure consistency and limit the vast array of methodological variations that arise when pooling inferences or data across studies. Multimodal assessments including volumetric and shape comparisons along with more advanced imaging assessments with diffusion MRI and connectomics may uncover microstructural differences and patterns of network organization more closely associated with behavioral measures and risk factors for disease. Sex differences with large effect sizes are rare in neuroimaging, but evidence for interactions and subtle effects is plausible. Harmonized processing schemes across studies in large‐scale consortia, such as ENIGMA, may reveal consistent sex differences in the brain and help us to identify factors that modulate potential effects such as environmental exposures, social interactions, and endocrine and genetic factors.

## CONFLICT OF INTEREST STATEMENT

The authors have no conflicts of interests to declare.

## ROLE OF AUTHORS

NJ and PT jointly wrote this review.
